# Is this the end of dried blood spots as we know it?

**DOI:** 10.1002/ansa.202300006

**Published:** 2023-07-19

**Authors:** Trine Grønhaug Halvorsen, Léon Reubsaet

**Affiliations:** ^1^ Department of Pharmacy University of Oslo Oslo Norway

## Abstract

In 2017 integrated sampling and sample preparation for simplified liquid chromatography‐mass spectrometry analysis of proteins from dried blood spots were introduced. The concept, called smart samplers or smart sampling, enables proteolysis or affinity clean‐up, two common sample preparation steps in liquid chromatography‐mass spectrometric bioanalysis of proteins, to start at the moment of sampling. The idea is to utilize the time for sampling and drying to perform these time‐consuming and labour‐intensive steps. Hence, only a simplified sample preparation is necessary after the arrival of the sample in the lab. In this perspective, we present an overview of the smart sampling approach where the conventional protein analysis workflow is reshuffled to start already prior to arrival in the lab. In addition, we present a thorough discussion of integrating sample preparation steps such as proteolysis or affinity capture in the sampling. Finally, in the end, we try to answer the question if conventional dried blood spots will become obsolete in the future.

## 1 INTRODUCTION

Patient‐centric sampling is an approach where the collection of blood samples is made easier and more convenient for the patient without reducing the quality of the sample. In patient‐centric sampling, it is ideal if the patient her‐/himself can take the sample. Dried blood spot (DBS) and other microsampling techniques fulfil these criteria.^1^ DBS sampling has already successfully been applied in patient‐centric sampling for both therapeutic drug monitoring, clinical studies and biomarker analysis.^2^, ^3^, ^4^

Since many biomarkers are low‐abundance proteins, efficient and sensitive protein analysis from low‐volume samples (≤50 µl) such as DBS is important. Analysis of proteins from DBS has conventionally been performed by immunometric techniques; however, in recent years there is seen an increased use of liquid chromatography‐tandem mass spectrometry (LC‐MS/MS) for analysis of proteins from DBS.^5^, ^6^

A challenge with mass spectrometric protein analysis from DBS is that it requires extensive and time‐consuming sample preparation after arrival in the analytical laboratory. This is especially true when the target analyte is low‐abundance and the sample volume is low. For sensitive analysis of low‐abundance proteins, the proteins are commonly proteolyzed to peptides prior to analysis, and in addition, a selective enrichment step including affinity capture is often included^7^.

**FIGURE 1:**
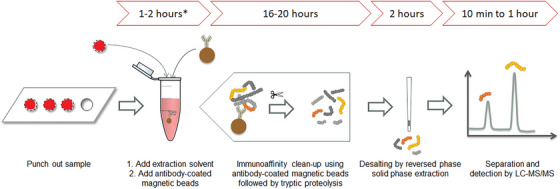
Overview of the common sample preparation steps and their time use in liquid chromatography‐tandem mass spectrometry (LC‐MS/MS) analysis of low abundant protein biomarkers from dried blood spot (DBS) after the arrival of the DBS in the lab. *Affinity capture may in some cases require overnight incubation. The affinity capture step can also be performed on the peptide level after the proteolysis step.

An overview of the common sample preparation steps and their time use in LC‐MS/MS analysis of low‐abundance protein biomarkers from DBS after arrival in the lab is shown in Figure 1. The main features of protein analysis from dried matrices are sampling – drying – transport (postal mail) – extraction/sample preparation – analysis. Especially the proteolysis step is time‐consuming. This is often performed *in‐solution* overnight (16–20 h) at a low enzyme‐to‐protein ratio to avoid autolysis.^8^ By immobilizing the proteolytic enzyme to a solid support efficient proteolysis in a shorter time can be achieved^8^, ^9^ as the enzyme‐to‐protein ratio can be increased without an increase in autolysis.^10^ The affinity‐capture step also adds time to the sample preparation. Most commonly, the capture is performed for up to a couple of hours at room temperature or 37°C,^11^, ^12^, ^13^, ^14^ but overnight capture at 4°C is also described.^15^, ^16^ Hence, the affinity‐capture step may also increase the handling time in the laboratory significantly. The sample preparation time comes in addition to the time needed for sampling, drying and transport. Hence, even though patient‐centric sampling is convenient for the patient it might result in increased response times in diagnostics and patient monitoring.

Based on all this we propose the following hypothesis: Will reshuffling of the sample workflow in protein analysis from DBS make conventional DBS analysis of protein analytes obsolete?

## 2 RESHUFFLING THE WORKFLOW IN MASS SPECTROMETRIC PROTEIN ANALYSIS FROM DBS

### 2.1 Why reshuffle?

In 2017 we introduced integrated sampling and sample preparation for the first time^17^. This was a fundamental and groundbreaking change in the typical workflow for protein analysis from dried matrices. The idea was to utilize the time for sampling, drying and transport to carry out as much sample preparation as possible. In this way, the workflow after receiving the sample in the analytical laboratory is simplified.

By modification of the sampling paper, commonly performed sample preparation steps in mass spectrometric protein analysis can be integrated into the sampling. This allows the instantaneous start of sample pre‐treatment when the droplet of blood is applied to the paper. As a result, only simple and short extractions are needed for analysis upon arrival at the laboratory, thus improving the response time drastically.

### 2.2 Which sample preparation steps can be integrated?

In mass spectrometric determination of low‐abundance proteins integration of either proteolytic enzymes or antibodies for affinity capture will make a difference in the sample preparation workload after arrival of the sampler in the laboratory. Both allow the time‐consuming sample preparation already to start at the moment of sampling.

#### 2.2.1 Proteolysis samplers

**FIGURE 2:**
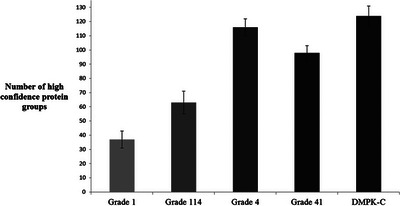
Number of high‐confidence protein groups identified from freshly collected whole blood samples (*n* = 3). For the analyses, pHEMA‐VDM polymerized Whatman grade 1, grade 114, grade 4, grade 41 and DMPK‐C immobilized with 1.25 mg trypsin (per spot) at room temperature for three hours. Reproduced from Skjærvø et al.^20^ with permission from the Royal Society of Chemistry.

Proteolytic samplers have been prepared by integration of the proteolytic enzyme trypsin into the sampling paper.^18^, ^19^, ^20^, ^21^ Various paper qualities (Table 1) have been investigated for trypsin immobilization and increased paper weight and thickness show improved digestion performance.^20^ This is illustrated in Figure 2. The reason for this may be due to the more homogenous polymer coating obtained using the thicker paper qualities as well as the thicker cellulose fibres seen for the heavier papers.^20^ The described proteolysis samplers are mainly evaluated for proteomics purposes reporting the number of proteins found in the digestion of a complex matrix. Both whole blood (undiluted)^20^ and serum (10‐fold diluted)^18^, ^19^ have been applied to the trypsin samplers. Better digestion (higher number of proteins identified) was obtained in diluted serum compared to whole blood: 199 protein groups were found in diluted serum digested on Grade 1 trypsin samplers^19^ compared to 57 in undiluted whole blood Grade 1 trypsin samplers.^20^ This is not unexpected. However, even more, efficient digestion is necessary if the samplers are to be a viable alternative to other available digestion methods. This may be obtained by using a thicker paper as shown for the Grade 4 and DMPK‐C cards above. Part of the reason for the rather modest protein number may be that the conventional workflow is reshuffled: reduction and alkylation are performed after digestion and not before.

The reshuffled workflow with post‐proteolysis reduction and alkylation has been compared to conventional pre‐proteolysis reduction and alkylation on a set of model proteins.^17^ Here mainly the same peptides were found using both methods, although certain proteins favoured either pre‐ or post‐reduction and alkylation. Based on the results it was concluded that the normal workflow could be reversed and the reduction and alkylation performed post proteolysis.^17^ In general it strongly depends on the nature of the proteins/peptides to be monitored if reduction and alkylation are needed. We recommend performing the reshuffled workflow with post‐proteolysis reduction and alkylation if cysteine‐containing peptides are to be monitored. Post‐proteolysis reduction and alkylation can also be advantageous in order to avoid blockage through large protein fragments in the downstream analysis.

The advantages of the trypsin sampler are the low degree of autolysis and the sampler's stability. Using Grade 1 filter paper and periodate oxidation to produce the trypsin samplers the autolysis was shown to be less than 2% of the autolysis seen for overnight digestion in solution. A low degree of autolysis is a common feature of immobilized trypsin^8^ here also indicating the successful immobilization onto the paper. Additionally, the trypsin sampler did not show a significant decrease in activity over a period of 4 months (stored dry at 4°C).^18^

Trypsin samplers have also briefly been investigated for use in targeted protein determination. Serum spiked with the biomarker progastrin‐releasing peptide (ProGRP) was added to trypsin samplers and subsequently, the ProGRP‐specific peptide ALGNQQPSWDSEDSSNFK was isolated using peptide affinity capture.^21^ Satisfactory linearity (R^2^ = 0.99) and repeatability (relative standard deviation [RSD] ≤ 16% at all levels except at the limit of quantification which showed RSD = 26%) were obtained. The limit of detection (LOD) was in the same range as previously seen for ProGRP from serum using peptide affinity capture (150 pg/ml vs. 54 pg/ml^22^ and 520 pg/ml^23^). This indicates that the proteolysis samplers also are suitable for use in quantitative determination of proteins.

**TABLE 1:** Commercially available laboratory filter paper tested by Skjærvø et al.^20^ for the fabrication of trypsin samplers. All filter papers are branded under GE Healthcare Whatman. All specifications are collected from the manufacturer. The table is reproduced and adapted from Skjærvø et al.^20^ with permission from the Royal Society of Chemistry.

	**Grade 1**	**Grade 4**	**Grade 41**	**Grade 114**	**DMPK‐C** ^1^
Thickness (µm)	180	205	220	190	N.A.
Pore size (µm)	11	20–25	20–25	25	N.A.
Weight (g/m^2^)	87	92	85	77	N.A.
Filtration speed (s per 100 ml)	150	37	54	38	N.A.

^1^ Specifications not available from the manufacturer. Skjærvø et al.^20^ report DMPK‐C to be approximately twice as thick as the other papers.

N.A. Not available.

#### 2.2.2 Affinity samplers

Integration of antibodies into the paper samplers is described to less extent than the proteolysis samplers. However, a couple of examples are available. Both of them describe the integration of a monoclonal antibody targeting the cancer marker and doping agent human chorionic gonadotropin (hCG).^24^, ^25^ The samplers were applied to both serum^24^, ^25^ and whole blood^24^ samples. A higher volume of serum could be applied to the samplers due to the lower viscosity and higher water content (40 vs. 30 µl^24^). The serum sample also efficiently wicked into the sampler while the slow wicking of the whole blood sample resulted in a dried blood crust on top of the paper surface. This might mainly be due to the paper modification procedure used for antibody immobilization, which created a less polar surface, as well as the fact that a rather large sample volume was used compared to the paper size.^24^ Different procedures for antibody immobilization may reduce this effect. The quantitative performance of the antibody samplers was evaluated both for thin paper (Grade 1) using a 20 µl sample and thick paper (DMPK‐C) using a 40 µl sample. The thicker paper sampled with 40 µl serum performed the best (better correlation and overall lower SDs).^24^ They also showed better correlation and comparable RSD to what had been observed previously for hCG spiked serum samples added to conventional DMPK‐C material and subsequently cleaned‐up using magnetic‐bead affinity capture and solid‐phase extraction.^11^, ^24^ However, a more thorough comparison using fully validated methods is needed to determine how the affinity sampler approach performs compared to the conventional lab approach.

#### 2.2.3 Bioaffinity tag samplers

**FIGURE 3:**
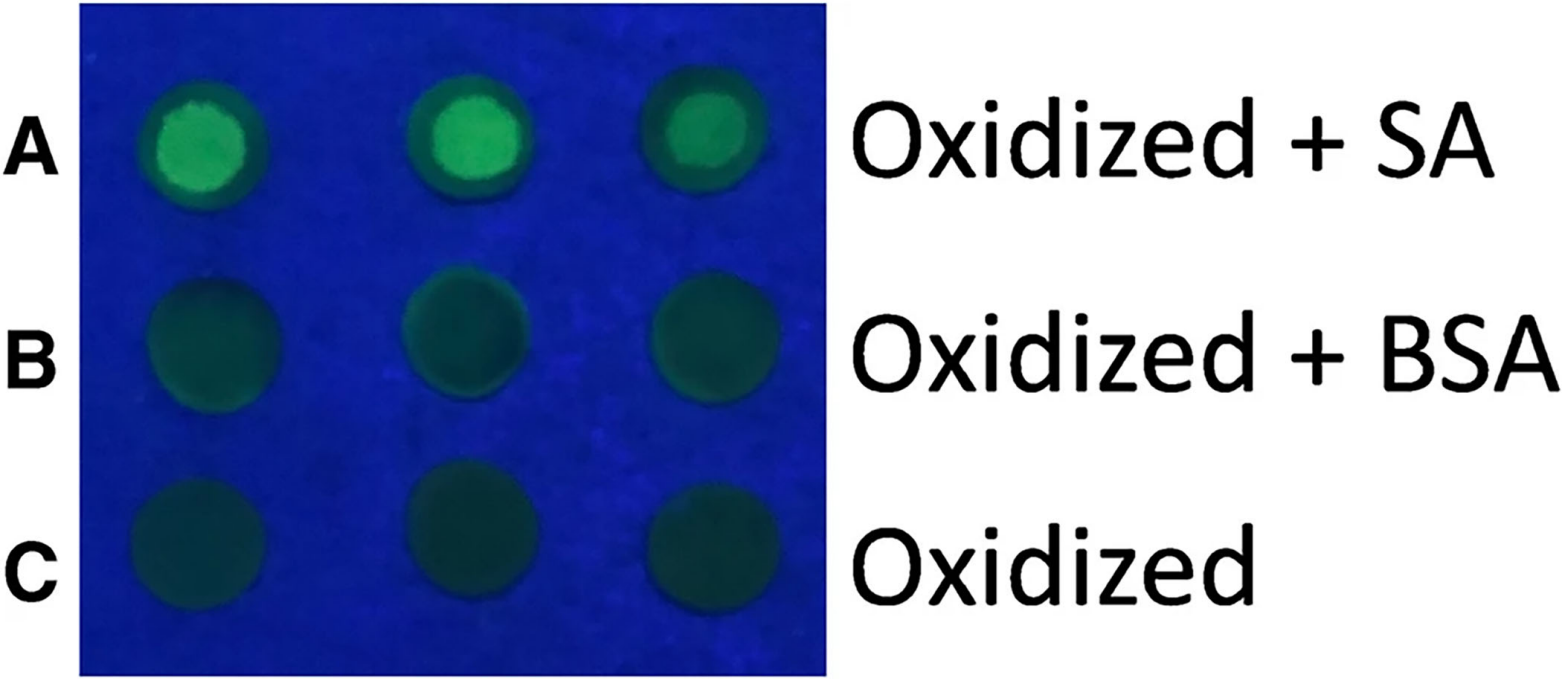
Photograph of paper discs under UV light (254 nm, *n*  =  3): (A) oxidized paper treated with 2% (w/v) 0.5 µl streptavidin (SA) and incubated with biotin‐5‐fluorescein (B5F), (B) oxidized paper treated with 0.5 µl 2% (w/v) bovine serum albumin (BSA) and subsequent incubation with B5F, and (C) oxidized paper discs directly incubated with B5F. Reprinted with permission from Johannsen et al.^26^

The proteolysis samplers and affinity samplers described above are premade and ready to use. This is very convenient for the end user. Still, in some cases, a universal sampler that easily can be adapted to the required application by the end user him‐/herself may be of interest. This can be obtained by immobilization of a bioaffinity tag such as streptavidin (SA) to the paper. After immobilization of the bioaffinity tag the bioaffinity system can be used to create the desired sampler: If streptavidin is immobilized the biotin‐SA system can be used to produce either a proteolysis sampler using biotinylated enzymes or an affinity sampler using biotinylated antibodies. The final immobilization of biotinylated enzyme or antibody can then be done on‐site by the end user. One example of a streptavidin sampler applied in MS analysis of proteins is available in the literature.^26^ Here streptavidin is immobilized on periodate oxidized paper, and in a second step the affinity samplers are prepared by a short (30 min) incubation of a biotinylated antibody on the SA sampler. The success of streptavidin immobilization was proven using a fluorescent tag (Figure 3), where only the samplers incubated with SA emitted strong fluorescence after incubation with biotin‐5‐fluorescein (B5F). Subsequently, incubation of SA samplers with biotinylated antibodies provided efficient capture of the target protein hCG from serum. This was in contrast to samplers without SA or where the SA was blocked with biotin or a non‐biotinylated antibody was added. These were showing low to modest signals (≤17.7%) compared to the signal intensity for SA samplers incubated with biotinylated antibodies.^26^ The quantitative performance of the SA‐biotin‐anti hCG samplers was satisfactory with RSDs ≤ 9.1% (200–1000 pg/ml), R^2^ = 0.981 (polynomial regression) and an LOD of 65 pg/ml from 5 µl of spiked serum.

#### 2.2.4 Integration of other functionalities in the sampler

For use in protein analysis by MS integration of proteolytic enzymes and antibodies, as well as integration of bioaffinity tags for the simple production of proteolytic enzyme of antibody samplers will make an impact on the sample preparation after arrival in the laboratory. However, it is also possible to integrate other functionalities in the sampling paper to facilitate the sample preparation to start at the moment of sampling. Some features have already been integrated into the paper for protein analysis but not in combination with MS analysis. Molecularly imprinted polymers (MIPs) is one example. MIPs are often referred to as synthetic antibodies. They are polymers with molecular recognition properties, tailor‐made to bind a specific analyte.^27^ MIPs are most commonly used in sample preparation of small molecules, but also MIPs for selective enrichment of proteins are described.^27^ Several MIPs, including MIPs targeting proteins, have been imprinted on paper.^28^ These are used mainly used in sensing systems, e.g., MIPs for chymotrypsinogen determination in urine using a distance detection‐based microfluidic paper^29^ and a fluorescent MIP for ferritin determination.^30^ However, MIPs for the capture of small molecules (metabolites) from urine have been polymerized on paper for analysis by paper spray MS.^31^ Hence, the production of MIPs on paper for protein analysis by MS should also be feasible. Subsequently, these paper MIPs could be incorporated into the sample workflow similarly to affinity samplers.

Other paper‐based sorptive phases immobilized on paper are also described.^32^ None of them is as specific for protein analysis as antibodies and proteolytic enzymes, but, e.g., ZrO_2_‐coated paper for the capture of diol‐containing compounds has been described.^33^ This can be used for the capture of glycoproteins or ‐peptides prior to MS analysis.

### 2.3 Paper modification strategies

**FIGURE 4:**
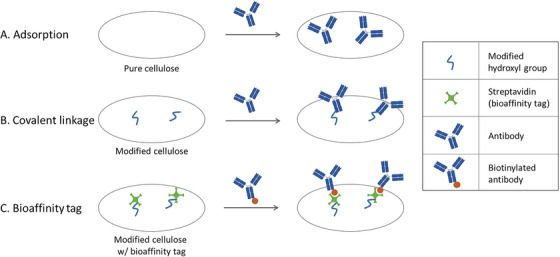
Schematic illustration of different immobilization strategies: (A) adsorption, (B) covalent linkage and (C) through the use of bioaffinity tag. The illustration shows the immobilization of antibodies, but the approaches can also be used for immobilization of enzymes (e.g. trypsin).

Biomolecules can be integrated into the DBS sampler either through adsorption, via a bioaffinity tag or linked through covalent binding^34^ (Figure 4). Adsorption is possible without any modification of the paper surface, however, in general proteins (e.g. enzymes and antibodies) are not strongly attached to pure cellulose.^34^ In addition, the non‐covalent interaction between proteins and paper is sensitive to changes in pH, ionic strength etc. Hence, a stronger immobilization strategy is commonly needed to efficiently attach the proteins to the paper surface. When coupling enzymes or antibodies through a bioaffinity tag, the bioaffinity tag (e.g. protein A/G or streptavidin) is first attached to the paper surface. This tag can be coupled to the paper similarly to when the enzyme or antibody is covalently linked to the paper; by modification of the hydroxyl groups on the paper surface. Different modifications of the cellulose hydroxyl groups have been investigated; e.g., oxidation into a carboxylic acid and subsequent carboxyl acid activation by N‐ethyl‐N′‐(3‐(dimethylamino)propyl)carbodiimide/N‐hydroxysuccinimide (EDC/NHS),^35^, ^36^ oxidation into aldehydes^18^, ^19^, ^36^ and epoxides,^35^ and silanization followed by subsequent 2‐hydroxyethyl methacrylate‐co‐2‐vinyl‐4,4‐dimethyl azlactone (HEMA‐VDM) polymerization^20^, ^21^, ^24^, ^25^ or HEMA‐tosylation.^25^ In combination with MS coupling through both covalent linkage^18^, ^19^, ^20^, ^21^, ^24^, ^25^ and bioaffinity tag^26^ is described. In addition, adsorption is briefly evaluated.^25^ However, as described above adsorption of proteins to cellulose is in general poor. This was also seen for the hCG‐specific antibody E27 where the adsorbed antibody showed a significantly lower ability to capture hCG from serum samples than antibodies covalently linked to the HEMA‐VDM or HEMA‐tosyl polymer (less than 8% signal intensity was seen for samples applied to paper with adsorbed antibody.^25^

### 2.4 Effect of sampler modification on sampling performance

The above‐mentioned modification strategies affect the paper structure differently. Adsorption is assumed to affect the paper structure least as the paper itself is not modified. With respect to paper structure, silanization and subsequent polymerization with HEMA‐VDM or HEMA‐tosyl makes the paper more rigid^20^ compared to when using periodate oxidation to produce aldehyde groups where the proteins can be covalently attached through a Schiff base. For the latter, the paper properties are retained (unpublished data). We believe retaining the paper properties is important in order to make smart samplers attractive for the end user.

One presumed disadvantage of the periodate oxidation strategy is the reversibility of the Schiff base that is produced. The stability can be improved by NaCNBrH_3_‐mediated reduction of the Schiff base.^37^ However, it has been seen that the reduction might lead to less efficient trypsin samplers compared to both HEMA‐VDM polymerized discs as well as non‐reduced periodate oxidized samplers.^19^

Díaz‐Liñán et al.^32^ describe the modification process of paper to be problematic in some cases due to the slow diffusion of the reagents from the bulk solution to the paper surface. Their suggestion for this limitation is to use micro‐ or nano‐cellulose as the precursors instead of paper as these materials can easily be dispersed in the reaction medium, guaranteeing a good functionalization. Once the cellulose has been modified, it can be transformed into a planar substrate by simple deposition.^32^ This may be an advantage also in the production of functionalized samplers for mass spectrometric determination of proteins.

### 2.5 Modification of other microsampling devices

**FIGURE 5:**
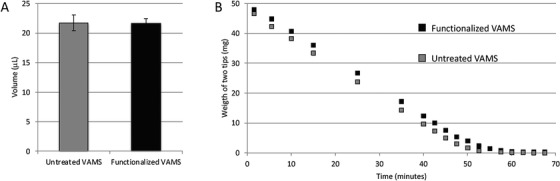
(A) Volume measurement of untreated VAMS and functionalized VAMS tips (n = 3). (B) Drying speed of untreated and functionalized VAMS tips. Weighing was carried out with two 20 µl tips at the same time. Reprinted with permission from Reubsaet et al.^38^

To our knowledge, modification of the volumetric absorptive microsampler (VAMS) by integration of the proteolytic enzyme trypsin to the sampling material^38^ is the only description of a modification of other microsampling devices than the paper‐based. VAMS is a microsampler capable of capturing a constant volume of the biological matrix. It has become a valuable alternative to filter paper in microsampling for bioanalytical purposes,^39^ and has been used in combination with MS both for the quantitative determination of proteins^40^, ^41^, ^42^ as well as proteomics purposes^43^. The efficiency of the trypsin VAMS sampler was described to be similar to the paper‐based proteolysis samplers with nearly 100 identified proteins from a diluted serum sample.^18^, ^19^, ^20^ In addition, the modified VAMS retains its property to absorb a defined volume of the sample as well as the drying speed after modification (Figure 5).^38^

### 2.6 Preparation of smart samplers

The use of smart samplers will free time and labour after the arrival of the samples in the laboratory. In addition, the estimated costs of smart sampler production are modest. The chemicals needed for paper functionalization before trypsin or antibodies are immobilized are of low costs (≪ 1 euro cent per sampler). For the preparation of the proteolysis samplers, approximately four times more trypsin is needed per sample compared to the amount needed for an in‐solution digest of a serum sample. For the affinity samplers, the amount of antibody per sampler is comparable to the amount needed for affinity capture by magnetic beads. As the proteolytic enzyme trypsin is less expensive than monoclonal antibodies the estimated cost of the proteolysis sampler is one‐fifth of the affinity sampler. Nevertheless, the cost estimate for both samplers is less than 1 € per disc/sampler.

## 3 CONCLUDING REMARKS

The answer to the question, of whether conventional DBS analysis of protein analytes will become obsolete, is not an easy one to give. At the present moment, conventional DBS/microsampling will still be the sampling method of choice for less challenging analytes (including proteins) and applications.

The main improvement of smart samplers to the DBS workflow of protein analysis is the possibility of starting the necessary pretreatments already at the time of sampling. This may result in a more time‐efficient analysis when the sample arrives in the laboratory. In addition, smart samplers may increase the specificity of the analysis. This latter especially is of importance in the determination of low‐abundance analytes.

Currently, only research applications are available using smart samplers. In order to fully evaluate the potential of the concept the production needs to be optimized and streamlined. In addition, validated methods using both proteolysis samplers and affinity samplers need to be developed and the samplers must be evaluated on a larger set of real samples.

Although the smart samplers may not make conventional DBS analysis of protein analytes obsolete, we believe the samplers will become a valuable addition to challenging analytes and niche applications. They will for instance have a potential in proteomics application from small volume samples both by initiating the proteolysis at the moment of sampling as well as in selective capture of low abundant analytes from biological matrices.

## CONFLICT OF INTEREST STATEMENT

The authors declare no conflict of interest.

## Data Availability

Data sharing is not applicable – no new data were generated.
